# Supplementary material for: Mechanisms governing the pioneering and redistribution capabilities of the non-classical pioneer PU.1
Source: Nat Commun. 2020 Jan 21;11:402. doi: 10.1038/s41467-019-13960-2 (PMC6972792; doi:10.1038/s41467-019-13960-2)
Supplement: Supplementary file 7 — Source data [file 41467_2019_13960_MOESM7_ESM.zip › Source_Data/FigureS2/FigureS2B_MotifScanOutput/KmeansCluster2/homerResults/motif2.similar.html]

motif2

## Information for motif2

T
C
G
A
T
G
A
C
A
C
T
G
C
T
G
A
A
T
C
G
T
A
C
G
C
G
T
A
C
G
T
A
C
T
A
G
  
Reverse Opposite:  

A
G
T
C
G
C
A
T
A
C
G
T
A
T
G
C
A
T
G
C
G
A
C
T
A
G
T
C
A
C
T
G
A
G
C
T
  

|  |  |
| --- | --- |
| p-value: | 1e-914 |
| log p-value: | -2.107e+03 |
| Information Content per bp: | 1.708 |
| Number of Target Sequences with motif | 2620.0 |
| Percentage of Target Sequences with motif | 52.16% |
| Number of Background Sequences with motif | 4608.9 |
| Percentage of Background Sequences with motif | 11.34% |
| Average Position of motif in Targets | 100.9 +/- 51.1bp |
| Average Position of motif in Background | 104.1 +/- 83.2bp |
| Strand Bias (log2 ratio + to - strand density) | 0.0 |
| Multiplicity (# of sites on avg that occur together) | 1.04 |
| Motif File: | file (matrix) reverse opposite |

### Similar de novo motifs found

|  |  |  |  |  |  |  |  |
| --- | --- | --- | --- | --- | --- | --- | --- |
| Rank | Match Score | Redundant Motif | P-value | log P-value | % of Targets | % of Background | Motif file |
| 1 | 0.824 | A G C T A C G T G C A T A G T C A G T C A G C T A T G C | 1e-863 | -1988.147742 | 74.32% | 28.48% | motif file (matrix) |
| 2 | 0.790 | A T G C A G C T A G C T A T G C A G T C A G C T A G T C G A C T A G C T | 1e-644 | -1483.627026 | 36.95% | 7.25% | motif file (matrix) |
| 3 | 0.766 | C T A G T C G A C T G A T C A G T C G A C T A G A T C G T C G A C G T A T A C G T G A C T G C A | 1e-200 | -461.852244 | 20.98% | 6.72% | motif file (matrix) |
| 4 | 0.790 | C T A G C T G A C T A G A T C G C G T A C T G A C T A G T A G C C G T A C T A G | 1e-101 | -232.933032 | 9.72% | 2.78% | motif file (matrix) |
